# Perturbation of p38α MAPK as a Novel Strategy to Effectively Sensitize Chronic Myeloid Leukemia Cells to Therapeutic BCR-ABL Inhibitors

**DOI:** 10.3390/ijms222212573

**Published:** 2021-11-22

**Authors:** Yi-Hue Kuo, Shih-Hsiang Wei, Jie-Hau Jiang, Yueh-Shih Chang, Mei-Yin Liu, Shu-Ling Fu, Chi-Ying F. Huang, Wey-Jinq Lin

**Affiliations:** 1 Institute of Biopharmaceutical Sciences, College of Pharmaceutical Sciences, National Yang Ming Chiao Tung University, Taipei 112, Taiwan; lily08248277@gmail.com (Y.-H.K.); wei000068@gmail.com (S.-H.W.); cl4cl4123@yahoo.com.tw (J.-H.J.); lsnancy51@gmail.com (M.-Y.L.); cyhuang5@nycu.edu.tw (C.-Y.F.H.); 2 Hemato-Oncology, Department of Internal Medicine, Chang Gung Memorial Hospital, College of Medicine, Keelung & Chang Gung University, Taoyuan City 33302, Taiwan; yshichang@gmail.com; 3 Institute of Clinical Medicine, College of Medicine, National Yang Ming Chiao Tung University, Taipei 112, Taiwan; 4 Institute of Traditional Medicine, National Yang Ming Chiao Tung University, Taipei 112, Taiwan; slfu@nycu.edu.tw

**Keywords:** chronic myeloid leukemia, combined therapy, imatinib, dasatinib, p38 MAPK

## Abstract

Chronic myeloid leukemia (CML) is a hematopoietic malignancy characterized by the presence of the BCR-ABL oncogene. Therapeutic regimens with tyrosine kinase inhibitors (TKIs) specifically targeting BCR-ABL have greatly improved overall survival of CML. However, drug intolerance and related toxicity remain. Combined therapy is effective in reducing drug magnitude while increasing therapeutic efficacy and, thus, lowers undesired adverse side effects. The p38 MAPK activity is critically linked to the pathogenesis of a number of diseases including hematopoietic diseases; however, the role of each isozyme in CML and TKI-mediated effects is still elusive. In this study, we used specific gene knockdown to clearly demonstrate that the deficiency of p38α greatly enhanced the therapeutic efficacy in growth suppression and cytotoxicity of TKIs, first-generation imatinib, and second generation dasatinib by approximately 2.5–3.0-fold in BCR-ABL-positive CML-derived leukemia K562 and KMB5 cells. Knockdown of p38β, which displays the most sequence similarity to p38α, exerted distinct and opposite effects on the TKI-mediated therapeutic efficacy. These results show the importance of isotype-specific intervention in enhancing the therapeutic efficacy of TKI. A highly specific p38α inhibitor, TAK715, also significantly enhanced the imatinib- and dasatinib-mediated therapeutic efficacy, supporting the feasibility of p38α deficiency in future clinic application. Taken together, our results demonstrated that p38α is a promising target for combined therapy with BCR-ABL-targeting tyrosine kinase inhibitors for future application to increase therapeutic efficacy.

## 1. Introduction

Small-molecule tyrosine kinase inhibitors (TKIs) have profoundly improved the prognosis and life expectancy of various cancer patients since the first tyrosine kinase inhibitor imatinib was approved for clinical use to treat chronic myeloid leukemia (CML) [1]. However, these kinase inhibitors, even for those with one known prominent target, frequently act on multiple protein kinases and often results in adverse off-target side effects leading to undesired intolerance or toxicity, particularly upon high-dose and long-term treatment [2]. One of the strategies to combat this obstacle is combination therapy, which targets either TKI-related or TKI-unrelated survival pathways, death pathways, or feedback inhibitory/activating pathways, thus not only reducing drug doses but also providing additional therapeutic efficacy [3].

CML is a clonal hematopoietic disease characterized by the presence of the Philadelphia (Ph) chromosome caused by a reciprocal translocation between chromosomes 9 and 22 [4]. This leads to the formation of the BCR-ABL fusion gene and constitutive activation of the tyrosine kinase activity resulting in uncontrolled proliferation, survival, and dysregulated differentiation of myeloid cells [5]. The disease occurs prevalently in elderly people over the age of 55. This group of people is more fragile and frequently present underlying health conditions, which increase complications related to drug treatment and compromise outcomes [6].

The BCR-ABL oncogene activates multiple important signaling pathways, including Ras-MAPKs, STATs, SFKs (Src family kinases), and PI3K/AKT, to promote growth and survival [7,8,9]. The first BCR-ABL-targeted protein TKI, imatinib, has changed the treatment regimens for CML and resulted in significantly improved remission rate, prognosis, and overall survival [10]. Imatinib, with an unprecedented specificity toward the kinase activity of BCR-ABL, achieved a remarkable remission rate of 95% for CML patients in the chronic stage [11]. It works as a competitive inhibitor at the ATP binding site of BCR-ABL, which leads to inactivation of the kinase activity and results in the elimination of BCR-ABL-positive hematopoietic cells [10]. A complete eradication of CML is, however, yet to be achieved. Approximately 33% of CML patients do not achieve a complete cytogenic response (CCyR) after treatment [12]. The second-generation TKIs, such as dasatinib, show significant effects in patients resistant to imatinib; however, intolerance to the drug-related toxicity and off-target adverse effects leading to unnecessary discontinuation of treatment remain [13,14,15]. Studies on the development of potential novel strategies are needed to overcome these obstacles.

Understanding the molecular mechanisms through which BCR-ABL promotes leukemia generation and TKIs exert anti-leukemic effects will greatly facilitate the development of novel combined therapeutic strategies for clinical utility. It is presumed that imatinib and other TKIs targeting BCR-ABL oncogene exert their anti-leukemic effects by blocking BCR-ABL-driven mitogenic and anti-apoptotic signaling pathways. However, the precise role of each pathway is not completely elucidated. The p38 MAPK family is a group of versatile serine/threonine kinases. Dysregulation of the p38 MAPK activity is critically linked to the pathogenesis of hematopoietic disease, rheumatoid arthritis (RA), chronic obstructive pulmonary disease (COPD), and malignancy [16,17] and, therefore, the development of specific inhibitors for clinical purposes has attracted vast attention [18]. The four members of p38 MAPKs (i.e., p38α, p38β, p38γ, and p38δ) exhibit high sequence homology with either overlapped or distinct physiological and pathological roles [19]. The role of p38 MAPK in imatinib-mediated anti-leukemic effects is controversial. Some reports have shown that imatinib treatment induced the activation of p38 MAPK signaling pathways and affected the proliferation or apoptosis of BCR-ABL-expressing cells [20,21]. However, other studies have shown opposite results, where the inhibition of p38 MAPK with SB202190 did not affect imatinib-mediated caspase activation [22]. This may be, at least partly, due to the fact that the pharmacological inhibitors used do not discriminate well between isozymes in the p38 MAPK family, particularly p38α and p38β.

In this study, we elucidated the distinct roles of p38α and p38β in imatinib-mediated therapeutic effects by gene-specific knockdown. Our results clearly showed that deficiency in p38α greatly enhanced imatinib-induced therapeutic effects in growth suppression and apoptotic cell death in two BCR-ABL-positive CML-derived leukemia cells, K562 and KBM5. Knockdown of p38β did not sensitize cells to the therapeutic effect of imatinib but rather was more resistant to imatinib-mediated killing and growth suppression. Deficiency of p38α also enhanced the efficacy of second-generation TKI dasatinib. The potential clinic application of our results was further supported by similar observations using the p38α-specific inhibitor TAK715, which possesses a very high discrimination capability between the α and β isoforms. Taken together, this study provides strong evidence demonstrating that p38α is a promising target for combined therapy with BCR-ABL-targeted tyrosine kinase inhibitors to increase therapeutic efficacy.

## 2. Results

### 2.1. Knockdown of p38α Significantly Enhanced Imatinib-Induced Cytotoxicity in BCR-ABL-Positive K562 Leukemia Cells

**Figure 1:**
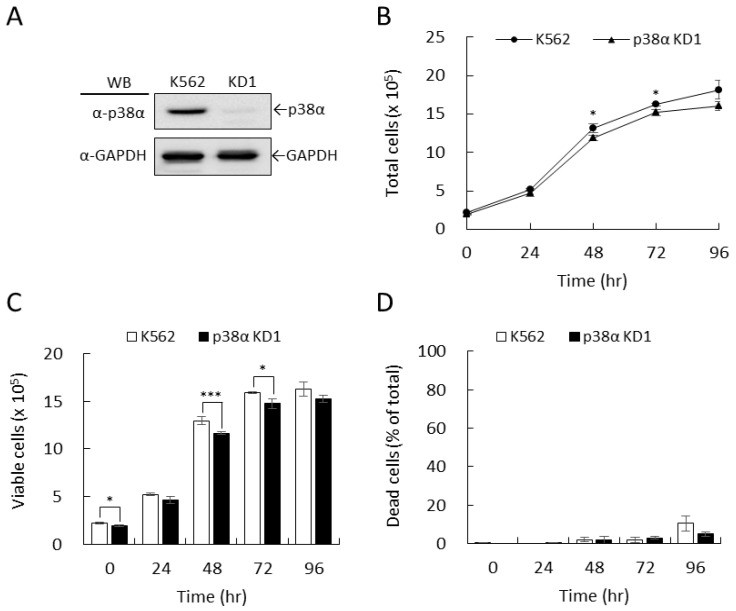
Knockdown of p38α had no apparent effects on the viability of BCR-ABL-positive K562 leukemia cells. The transcripts of p38α in K562 cells were knocked down using specific shRNAs. The protein levels of p38α were examined by Western blotting (**A**). The cell growth of parental K562 and knockdown KD1 cell clones were examined using hemocytometer counting under a microscope (**B**). The viable (**C**) and dead cells (**D**) were identified by trypan blue exclusion. All results shown are representatives of three independent experiments. Cell number and viability are presented as the mean ± SE of three repeats. * *p* < 0.05, and *** *p* < 0.005.

Members of MAPK pathways are reported to associate with imatinib-mediated effects [20,21,22]; however, the roles of each member in imatinib-mediated effects are still elusive. Our previous studies have revealed distinct roles for p38α and p38β in the differentiation of K562 cells by using specific gene knockdown [23,24,25]. We used the established p38α-knockdown stable clone KD1 of K562 cells (Figure 1A) in this study. The growth of knockdown cells was slightly slower, approximately 90%, as compared to parental K562 cells under regular culturing conditions (Figure 1B). The total viable cells of KD1 were approximately 90% compared to those of parental K562 (Figure 1C), which was parallel to the total cell number (Figure 1B). There was no difference in dead cell numbers between K562 and KD1 (Figure 1D). This indicated that the viability of KD1 cells was similar to the parental cells, suggesting knockdown of p38α in K562 cells has no apparent effects on the survival of K562 cells.

**Figure 2:**
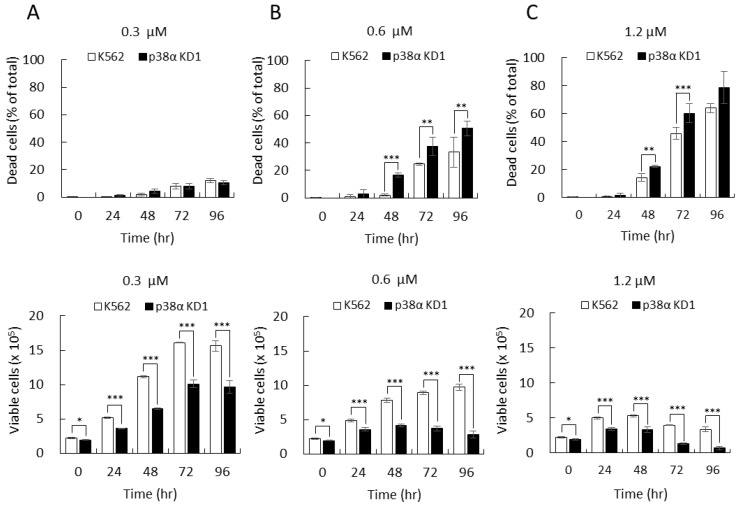
Knockdown of p38α significantly enhanced imatinib-induced cytotoxicity in BCR-ABL-positive K562 leukemia cells. The K562 parental and p38α-knockdown (KD1) cells were treated with 0.3 µM (**A**), 0.6 µM (**B**), and 1.2 µM (**C**) of imatinib. The viability of cells was examined by trypan blue exclusion. Knockdown of p38α greatly enhanced the therapeutic efficacy of imatinib. All results shown are representatives of three independent experiments. Total and dead cell numbers are presented as the mean ± SE of three repeats. * *p* < 0.05, ** *p* < 0.01, and *** *p* < 0.005.

We then investigated the responses of these cell clones to imatinib treatment. At most concentrations tested, imatinib exhibited a remarkable differential effect in K562 parental and p38α-knockdown cells. At 0.3 µM, imatinib only slightly affected cell viability, around 10% at 96 h, in both parental and KD cells (Figure 2A, dead cells); however, imatinib caused significant growth suppression in p38α-knockdown KD1 to approximately 60% of that of K562 parental cells (Figure 2A, 72 and 96 h). When the concentration was increased to 0.6 µM, imatinib exhibited remarkable cytotoxic killing in both cell clones with a much greater effect in the p38α KD cells. The dead cells increased to 25% and 33% in K562 parental cells at 72 and 96 h, respectively (Figure 2B, K562). In p38α-knockdown cells, the drug killed 37% and 51% cells at 72 and 96 h, respectively (Figure 2B, p38α KD1). The results showed that the imatinib-mediated killing increased by 1.5-fold when p38α was deficient. When growth suppression and cytotoxic killing were measured together, the overall differences in the therapeutic efficacy between p38α KD and parental K562 cells were further increased. The total viable cells of p38α KD1 were only 42% and 29% to those of the K562 parental cells at 72 and 96 h, respectively (Figure 2B, viable cells, 0.6 µM). These counted for an increase of 2.4-fold and 3.5-fold in therapeutic efficacy at 72 and 96 h, respectively, when p38α was deficient. At 1.2 µM, imatinib exerted a very strong therapeutic effect in K562 cells, as the viable cells decreased by 40–45% and 75–80% compared to those at 0.3 µM and 0.6 µM, respectively (Figure 2C). At this concentration (1.2 µM), imatinib still induced a much stronger response in p38α KD cells (Figure 2C). Similar results were observed with another p38α-knockdown KD11 cell clone in imatinib-mediated killing and cell growth suppression (Supplementary Materials Figure S1). The total viable cells represent a combined outcome of cell death and growth suppression as an overall measurement of therapeutic efficacy. These results provide strong evidence demonstrating that p38α deficiency greatly enhances the therapeutic efficacy of imatinib, particularly at the middle concentration of 0.6 µM, in BCR-ABL-positive leukemia K562 cells.

### 2.2. Imatinib Induced Apoptotic Cell Death in K562 Cells Which Was Significantly Enhanced When p38α Was Deficient

**Figure 3:**
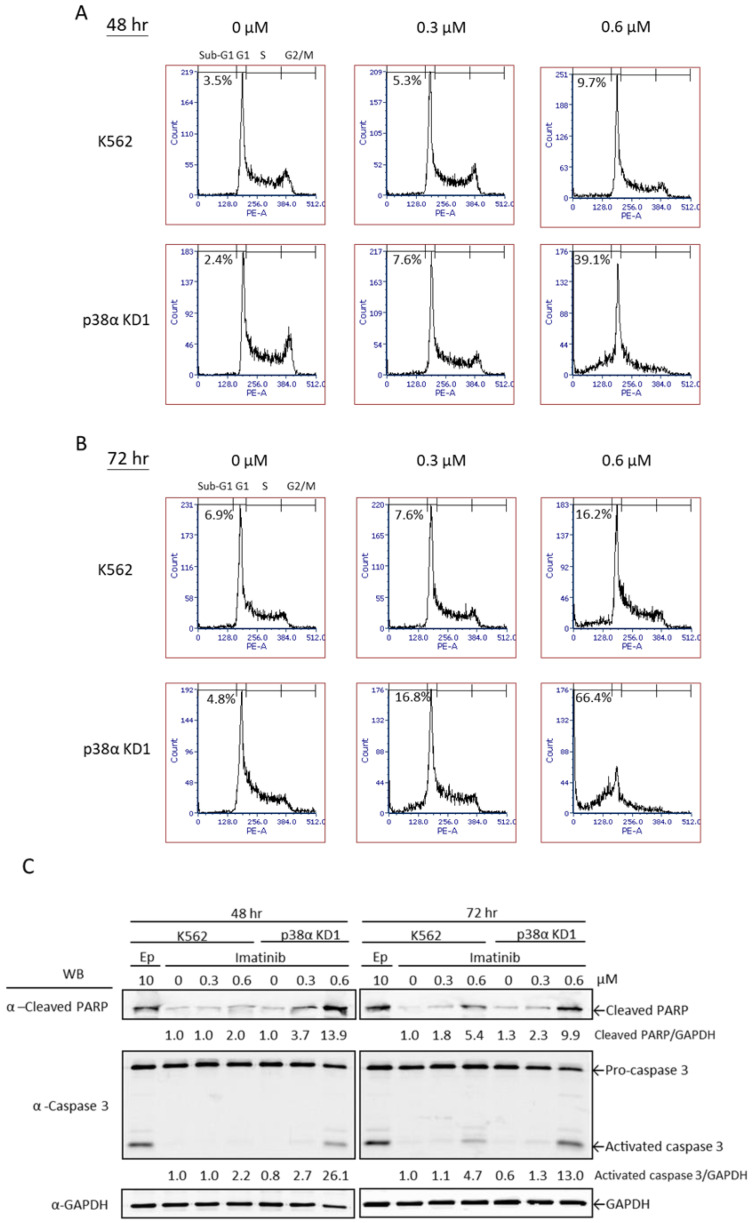
Imatinib-induced apoptotic cells death significantly increased when p38α was deficient. The K562 parental and p38α-knockdown (KD1) cells were treated with various concentrations of imatinib as indicated for 48 and 72 h. Cells were fixed and stained with propidium iodide. The cell cycle distribution was analyzed by flow cytometry: (**A**) 48 h and (**B**) 72 h. Knockdown of p38α significantly increased the apoptotic sub-G1 population after imatinib treatment. Activation of caspase-3 was analyzed by Western blotting using specific antibodies (**C**). Etoposide (Ep) is a chemotherapeutic drug known to induce apoptosis and used as a positive control. The ratio of band intensity was quantified as described in Section 4. Both the active form of caspase-3 and the cleavage of the specific substrate PARP increased in p38α knockdown cells. All results shown are representatives of three independent experiments.

We further investigated the molecular events involved in the increased cytotoxicity of imatinib toward p38α-knockdown cells. The parental and p38α-knockdown KD1 cells exhibited similar patterns in cell cycle distribution under normal culturing conditions (Figure 3A, 0 µM). At 0.3 µM, imatinib did not induce apparent change in either cell clones at 48 h (Figure 3A, 0.3 µM). As the concentration increased to 0.6 µM, imatinib induced a significant elevation in the sub-G1 population, a characteristic of apoptosis, to 39.1% in KD1 cells at 48 h, while it only resulted in 9.7% in the sub-G1 population of K562 cells (Figure 3A). When the duration of treatment increased to 72 h, imatinib (0.3 µM) caused a further increase in the sub-G1 population to 16.8% in p38α KD cells, but it did not appear to increase the sub-G1 population in K562 parental cells (Figure 3B, 0.3 µM). At 0.6 µM, imatinib caused a greater increase in the sub-G1 population to 66% in p38α KD cells. However, K562 cells exhibited only a slight increase in the sub-G1 population to approximately 16% (Figure 3A,B, 0.6 µM). These results indicate that imatinib caused a much greater extent of apoptotic cell death in p38α KD cells. Apoptosis was further examined with caspase activation by Western blotting analysis (Figure 3C). Caspase-3 is a major executioner caspase in apoptosis and is activated via cleavage of proenzyme [20]. The activated caspase-3 carries out a wide range of proteolytic actions on many important cellular proteins including PARP (poly ADP-ribose polymerase), a critical player in DNA repair. The cleavage of these two proteins thus provides good evidence indicating the progression of apoptosis. Deficiency of p38α significantly enhanced the extent of activation of caspase-3 and PARP cleavage in a shorter time period compared to parental K562 cells (Figure 3C). These results are consistent with those observed in the increase in the sub-G1 population and together validate the cytotoxicity effect and cellular viability observed by using the trypan blue exclusion assay (Figure 2). Etoposide (Ep) is a chemotherapeutic drug known to induce apoptosis and used as a positive control. The presence of the sub-G1 population, the activation of caspase-3, and the cleavage of PARP (Figure 3) are well-known characteristics of apoptosis. Taken together, these results indicate that imatinib causes apoptotic cell death in BCR-ABL-positive leukemia K562 cells and p38α deficiency significantly enhances this cytotoxic effect.

### 2.3. Knockdown of p38β Did Not Sensitize K562 Leukemia Cells to the Therapeutic Effects of Imatinib

**Figure 4:**
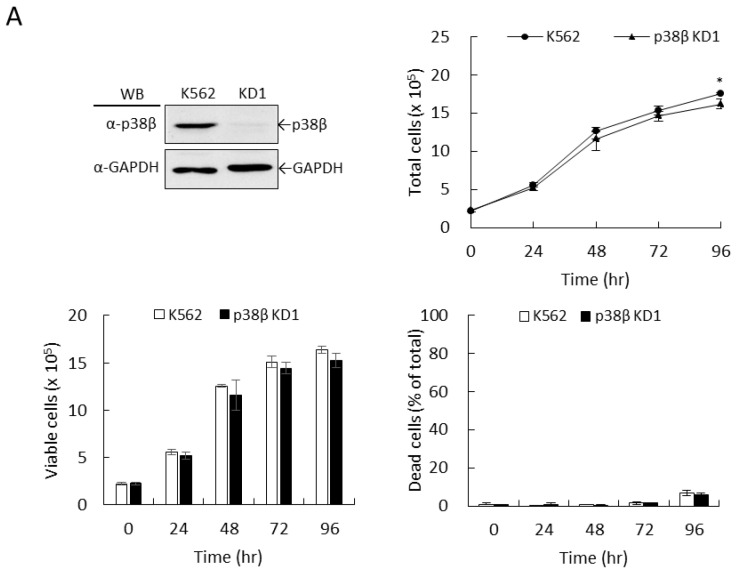

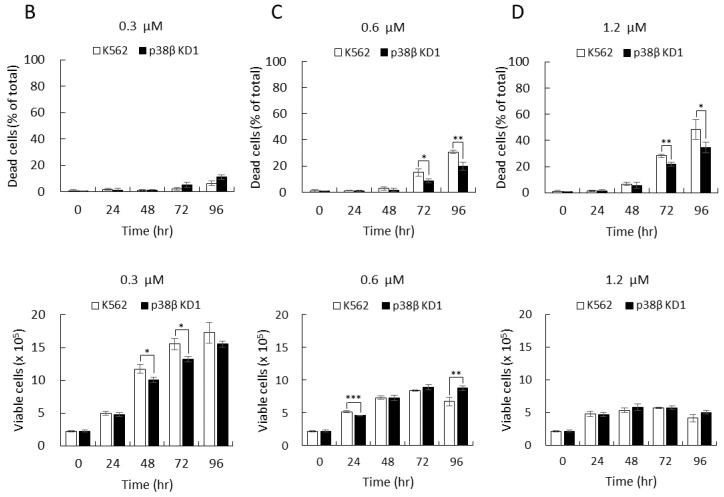
Knockdown of p38β did not sensitize K562 leukemia cells to the therapeutic effects of imatinib. The transcripts of p38β were knocked down using specific shRNAs. The protein levels of p38β were detected by Western blotting (**A**). The cell growth and viability of parental K562 and knockdown KD1 cell clones under normal culturing conditions were examined by trypan blue exclusion using hemocytometer counting under a microscope (**A**). Cells were treated with 0.3 (**B**), 0.6 (**C**), and 1.2 µM (**D**) of imatinib and the cell viability was examined. Deficiency of p38β did not sensitize K562 cells to the killing effect of imatinib as observed with p38α deficiency. On the contrary, p38β deficiency exhibited a slight but significant resistance to the killing effect of imatinib. All results shown are representatives of three independent experiments. Viable and dead cell numbers are presented as the mean ± SE of three repeats. * *p* < 0.05, ** *p* < 0.01, and *** *p* < 0.005.

The α and β isotypes of p38 MAPK exhibit the most similarity in sequence homology and regulation of enzymatic activity and are the most abundant members in many tissues [19]. However, many pharmacological inhibitors do not distinguish these two isozymes well, which hampers the progression in elucidating the individual role of these two isozymes in imatinib-mediated therapeutic effects. Therefore, we used a gene knockdown strategy to address whether p38β is involved in enhancing the cellular sensitivity of K562 to imatinib. The knockdown of p38β protein levels is shown in Figure 4A. The growth and viability of p38β-knockdown cells were similar to the parental K562 cells under normal culturing conditions (Figure 4B–D). Similar to what was observed with p38α knockdown (Figure 2A, upper), imatinib, at 0.3 µM, did not kill p38β-knockdown cells (Figure 4A, dead cells). Notably, growth suppression mediated by the imatinib treatment in p38β-knockdown cells was significantly less (Figure 4B, bottom) compared to p38α knockdown cells (Figure 2A, bottom), indicating the differential responses upon p38α and p38β deficiency. At higher concentrations, 0.6 and 1.2 µM, imatinib significantly caused cell death in K562 parental cells (Figure 4C,D, upper) as observed above (Figure 2). Notably, p38β deficiency did not render cells more sensitive to imatinib as p38α knockdown (Figure 2). On the contrary p38β KD cells were slightly more resistant to the killing (Figure 4C,D, upper) and the growth suppression (Figure 4C,D, bottom) mediated by imatinib as compared to parental cells.

### 2.4. The Cytotoxic Effect of Dasatinib Was Enhanced upon p38α Knockdown

**Figure 5:**
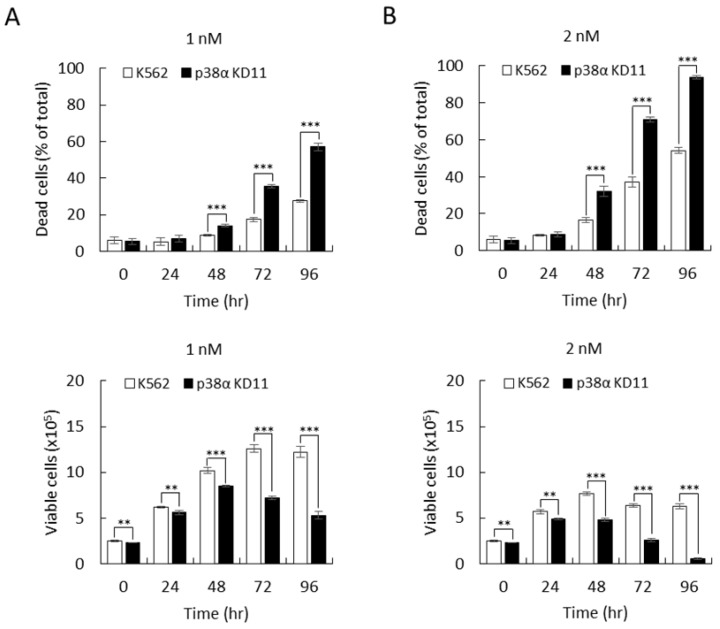
The cytotoxic effect of dasatinib was also enhanced upon p38α knockdown. The K562 parental and p38α-knockdown (KD11) cells were treated with 1 (**A**) or 2 nM (**B**) of the second-generation TKI dasatinib. The viability of cells was examined by trypan blue exclusion. Knockdown of p38α greatly enhanced the therapeutic efficacy of dasatinib. All results shown are representative of three independent experiments. Viable and dead cell numbers are presented as the mean ± SE of three repeats. ** *p* < 0.01, and *** *p* < 0.005.

Dasatinib is a second-generation BCR-ABL inhibitor with a higher inhibitory efficacy toward the kinase activity of the BCR-ABL oncogene [26] and is widely used to treat CML patients. Dasatinib binds to the active conformation of the kinase domain and also has a high inhibitory efficacy toward other tyrosine kinases, such as Src, and thus renders a higher risk of side effects [9]. We further investigated whether p38α might affect the killing effect of dasatinib. Dasatinib exerted a significant cytotoxic effect in K562 parental cells with 28% and 58% cell death at 1 nM and 2 nM, respectively (Figure 5A,B, 96 h, upper). The p38α-knockdown cells were much more sensitive to dasatinib. The cell death events were 57% and 93% at 1 and 2 nM, respectively (Figure 5A,B, 96 h, upper), which were almost double the death events in K562 parental cells. The total viable cells also reflected the profound increase in cellular responses to the killing effect of dasatinib (Figure 5A,B, bottom). Taken together, p38α deficiency greatly enhances the efficacy of two important, clinically used therapeutic TKIs—imatinib and dasatinib—in BCR-ABL-positive leukemia K562 cells. These results indicate a potential novel strategy in providing a more effective and safer treatment for CML by combining TKIs (e.g., imatinib or dasatinib) and specific p38α inhibition.

### 2.5. The p38α Deficiency-Mediated Drug Sensitivity Was Also Observed in BCR-ABL-Positive KBM5 Leukemia Cells

**Figure 6:**
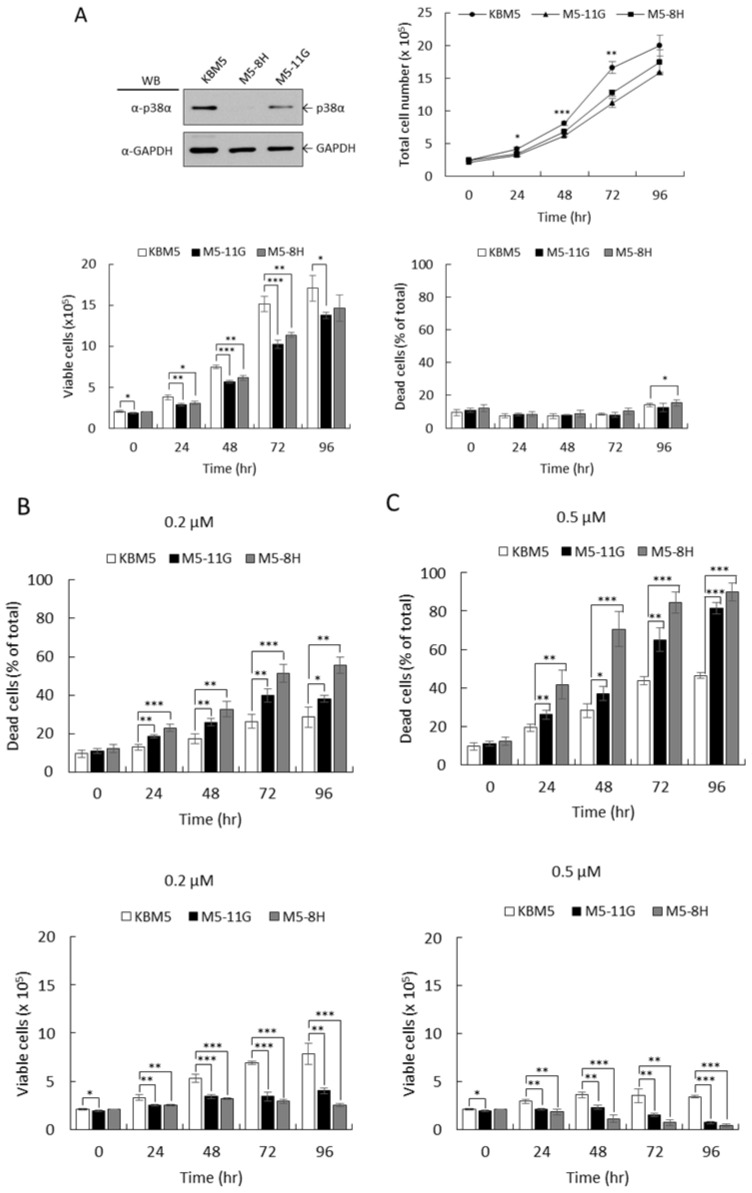
The p38α deficiency increased cellular sensitivity to imatinib in BCR-ABL-positive KBM5 leukemia cells. The p38α transcripts in KBM5 cells were knocked down using specific shRNAs. The protein levels of p38α were examined by Western blotting (**A**). The cell growth and viability of parental KBM5 and knockdown M5-8H and M5-11G cell clones were examined using hemocytometer counting under a microscope (**A**). Dead cells were identified by trypan blue exclusion. Cells were treated with 0.2 (**B**) and 0.5 µM (**C**) imatinib and the cell viability was examined. All results shown are representative of three independent experiments. Cell number and viability are presented as the mean ± SE of three repeats. * *p* < 0.05, ** *p* < 0.01, and *** *p* < 0.005.

KBM5 is a BCR-ABL-positive myelogenous leukemia cell line derived from a patient in the blast phase [27]. To investigate whether p38α deficiency-mediated drug sensitivity was also observed in other BCR-ABL-positive cells, we established the p38α-knockdown KBM5 stable clones M5-8H and M5-11G using shRNAs for investigation (Figure 6A). We carefully tested the drug concentrations for moderate cytotoxicity and chose 0.2 µM and 0.5 µM for the experiments. The growth of these knockdown cell clones was slightly slower compared to the parental KBM5 cells; however, the viability was similar (Figure 6A). These results are similar to those observed in K562 cells. The overall therapeutic efficacy of imatinib at 0.2 µM increased by 2.5–3.0-fold when p38α was knocked down (Figure 6B, viable cells). The killing also increased in p38α KD cells by 1.5–2.0-fold (Figure 6B, dead cells). At 0.5 µM, cell death greatly increased to 85% in p38-α knockdown cells compared to 46% dead events in parental cells (Figure 6C), which was an increase by approximately two-fold. These results were similar to those observed in K562 (Figure 2).

**Figure 7:**
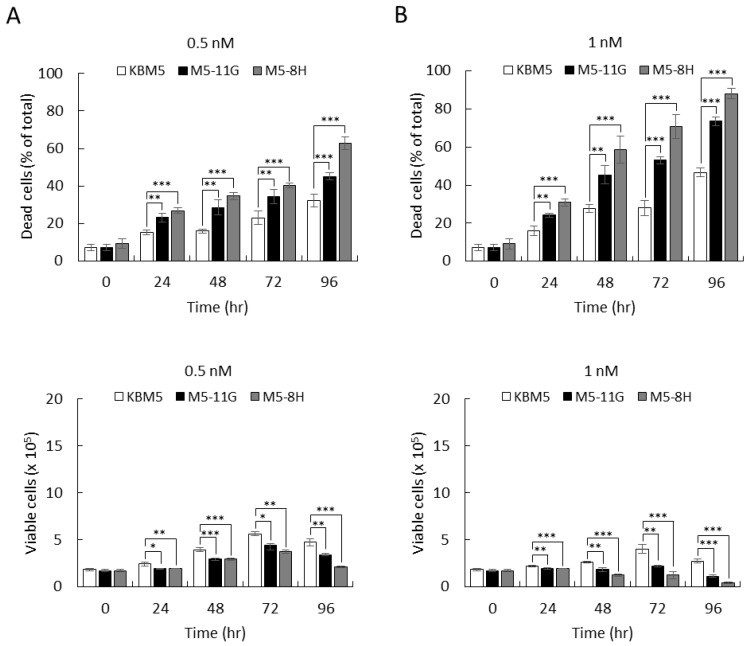
The p38α deficiency increased cellular sensitivity to dasatinib in BCR-ABL-positive KBM5 leukemia cells. The KBM5 parental and p38α-knockdown (M5-11G and M5-8H) cells were treated with 0.5 (**A**) or 1 nM (**B**) of second-generation TKI dasatinib. The viability of cells was examined by trypan blue exclusion. Knockdown of p38α greatly enhanced the therapeutic efficacy of dasatinib. All results shown are representative of three independent experiments. Viable and dead cell numbers are presented as the mean ± SE of three repeats. * *p* < 0.05, ** *p* < 0.01, and *** *p* < 0.005.

We further examined drug response to dasatinib. The results showed that cell death increased from 30% in parental cells to 45–60% in KD cells at 0.5 nM after 96 h (Figure 7A). At 1 nM, the death event increased to 45% in parental cells (Figure 7B) and increased to 70–90% in p38α KD cells (Figure 7B). At these effective drug concentrations, p38α deficiency greatly increased drug sensitivity by approximately two-fold. The cell viability presents a combined outcome of cell death and growth suppression and is used as an overall measurement of therapeutic efficacy. Our results together indicated that p38α deficiency-mediated sensitivity to therapeutic TKIs appeared to be common in BCR-ABL-positive CML cells and demonstrated that p38α is a promising target to lower drug dosing and increase the well-being of chronic myelogenous leukemia patients.

### 2.6. Specific p38α Inhibitor TAK715 Greatly Increased the Therapeutic Efficacy of Imatinib and Dasatinib toward CML Cells

**Figure 8:**
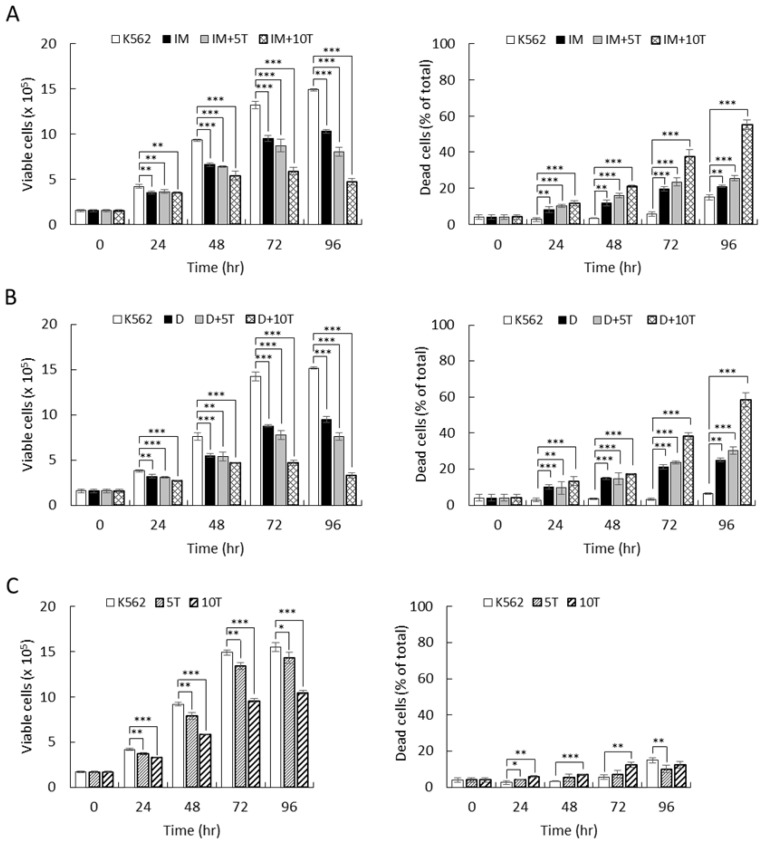
Specific p38α inhibitor TAK715 greatly increased the therapeutic efficacy of imatinib and dasatinib toward CML cells. K562 leukemia cells were treated with imatinib (0.3 µM) (**A**) or dasatinib (1 nM) (**B**) in combination with p38α inhibitor TAK715 (5 or 10 µM). Alternatively, cells were treated with TAK715 (5 or 10 µM) alone (**C**). The viability of cells was examined by trypan blue exclusion. All results shown are representative of three independent experiments. Viable and dead cell numbers are presented as the mean ± SE of three repeats. * *p* < 0.05, ** *p* < 0.01, and *** *p* < 0.005.

Due to the pivotal role of p38α in physiological and pathological processes, extensive efforts have focused on developing pharmacological inhibitors that can efficiently distinguish isozymes of the p38 family, particularly p38α and p38β [18]. TAK715 is a small molecule with an IC_50_ of 7.1 and 200 nM toward the α and β isoforms, respectively [28], and it exhibits a great capacity to distinguish the activity of these two isoforms. We therefore chose TAK715 to examine the response of CML cells to imatinib and dasatinib when the p38α activity was specifically inhibited by small molecular inhibitors, which have a more feasible application in clinic usage. TAK715 (10 µM) treatment, in combination with imatinib (0.3 µM), reduced the cell viability to 31%, whereas cells exhibited a much higher viability of 70% with imatinib treatment alone (Figure 8A, 96 h). The combined treatment also increased the cell death event from 20% (imatinib alone) to 53% (imatinib + TAK715) (Figure 8A, 96 h). These results suggest that with a combination including the p38α inhibitor TAK715, the therapeutic efficacy of imatinib is enhanced by approximately 2.3–2.7-fold. Similar effects, to a lesser extent, were also observed with a lower concentration of TAK715 at 5 µM (Figure 8A, IM+5T).

The combinatorial effect of TAK715 was further examined together with dasatinib. Results similar to those for imatinib were observed. Dasatinib (1 nM) treatment caused a reduction in cell viability to 63%. The viability was further reduced to 20% when cells were treated with dasatinib and TAK715 together (Figure 8B, D+10T, 96 h). The death events increased from 23% (dasatinib alone) to 58% (dasatinib + TAK715) (Figure 8B, D+10T, 96 h). These results indicate that the efficacy of dasatinib was enhanced by 2.5–3.2-fold when combined with TAK715. Similar effects, to a lesser extent, were also observed with a lower concentration of TAK715 at 5 µM (Figure 8B, D+5T). The treatment with TAK715 alone exhibited a growth suppression effect in a dose-dependent manner with 90% and 68% viable cells at 5 and 10 µM, respectively (Figure 8C, left); however, TAK715 alone had only mild effects on inducing cell death (Figure 8C, right). These results promise a feasible application for CML by combining p38 inhibition and imatinib/dasatinib treatment.

## 3. Discussion

The tyrosine kinase inhibitors targeting the BCR-ABL oncogene have profoundly changed the treatment regimen for CML by reaching sustained remission and prolonged survival. However, there are drawbacks to the use of these TKIs due to the fact of drug intolerance and undesired side effects after long-term usage [1]. In this study, we demonstrate that the therapeutic efficacy of two frontline drugs for CML, imatinib and dasatinib, was significantly enhanced when p38α activity was deficient upon gene knockdown or pharmacological inhibition. These results provide opportunities to lower drug doses and reduce side effects and, thus, shed light on a novel strategy for CML treatment through combination therapy.

Imatinib and dasatinib impede BCR-ABL-mediated aberrant pro-survival and mitogenic signaling [7,8]. Imatinib is the first TKI approved for clinic utility and greatly improves the remission rate, prognosis, and overall survival of CML patients [10]. However, there are approximately 15–25% non-responsive patients [12]. Adverse effects, such as pulmonary edema and congestive heart failure, leading to the discontinuation of treatment, develop after long-term drug treatment. The off-target effects and activation of BCR-ABL-independent pathways account, at least in part, for these obstacles [12]. Although imatinib was initially thought to target only BCR-ABL, it was later found to act on other kinases, including c-KIT and platelet-derived growth factor receptor (PDGFR), but not the Src family [10]. PDGFRβ is involved in regulation of fluid retention and may be one of the mechanisms for imatinib-associated pulmonary edema [15]. Dasatinib exhibits a much higher potency toward BCR-ABL inhibition [9]. Our results with K562 cells (Figure 1 and Figure 2) and KBM5 (Figure 6 and Figure 7) are consistent with these reports on the efficacy of these two TKIs. However, dasatinib is also more effective in inhibiting other kinases, such as the Src family of kinases (SFKs) and PDGFR, resulting in a higher toxicity and drug intolerance [15]. New strategies to lower drug concentrations while maintaining therapeutic efficacy are needed.

Adverse effects after TKI treatment are frequently observed. The underlying mechanisms include the off-target effect of kinase inhibitor, elicitation of multiple pathways of oncogenes, and aberrant activation of survival/downstream pathways upon drug treatment [1]. Satisfactory outcomes have been obtained through multiple pathway inhibition [3]. For instance, the combination of B-Raf inhibitor, dabrafenib (or vemurafenib), and MEK1/2 inhibitor, trametinib (or cobimetinib), for melanoma [29,30] and the combination of EGFR inhibitor and c-Met inhibitor for non-small cell lung cancer [31,32] have shown great improvement in therapeutic efficacy. MEK1/2 is a downstream effector of B-Raf, and EGFR shares several common downstream effectors with c-Met. These combination therapies have overcome drug resistance that arose due to the aberrant activation of downstream pathways. Possibilities to reduce the side effects associated with TKI treatment in CML by drug combination have been explored. Ponatinib, a third-generation TKI against BCR-ABL, is effective; however, with serious adverse effects such as cardiovascular disorders. A combination of ponatinib and forskolin, a natural chemical known to increase cellular cyclic AMP, shows a significant reduction in cell viability in CML cell lines [33]. The antiapoptotic BCL-2 family plays a key role in the survival of chronic myeloid leukemia. A combined treatment with a BCL-2 inhibitor and TKI markedly reduced CML cells and prolonged survival in a mouse CML model [34]. These results support that combination therapy has promising potential to reduce adverse side effects in CML.

The p38 MAPK pathway is one of the BCR-ABL-mediated downstream pathways [8]; however, their roles in the pathogenesis induced by BCR-ABL and in TKI-mediated therapeutic effects are still elusive. Imatinib treatment induces the activation of p38 MAPK signaling pathways in BCR-ABL-expressing CML-derived KT-1 cells [21] and K562 cells [35] as examined by Western blotting, suggesting a potential role in imatinib-mediated effects. Inhibition of p38 MAPK activity with pharmacological inhibitors SB203580 or SB202190, which inhibit both the α and β isoforms of p38 MAP, shows either reversion of imatinib-mediated cell growth suppression assayed by colony formation [21] or no apparent influence assayed by MTT assay [35]. These inconsistent results may arise due to the fact of various possibilities. In addition, the expression levels of these two isoforms in different cells vary to different extents. Therefore, the function of one isoform may be preferentially suppressed over the other one in a certain cell context, which leads to the controversial results. Dasatinib treatment is reported to induce activation of the p38 MAPK pathway in BCR-ABL-expressing cells [36]. Gene silencing using mouse MAPK14 (p38) *SMART*pool siRNA reverses dasatinib-mediated pro-apoptotic activity in mouse pro-B cell-derived BAF3/p210 cells and suppression of colony formation in CML KT-1 cells [36]. Our results show that knockdown of p38α enhances dasatinib-mediated cytotoxicity assayed by the trypan blue exclusion method (Figure 5). Further mechanistic investigation under various cell contexts and using various methods will be of great help in unveiling these differences. In this study, we clearly show that p38α deficiency enhances imatinib-mediated cytotoxicity and cell growth suppression in BCR-ABL-positive K562 and KBM5 cells (Figure 2, Figure 3, and Figure 6). A comparable therapeutic effect of imatinib, as measured by total viable cells, can be reached by using half amounts of drug in the p38α knockdown cells. The total viable cells of p38α KD1 were only 42% and 29% compared to those of K562 parental cells at 72 and 96 h, respectively (Figure 2B, viable cells, 0.6 µM). These counted for an increase of 2.4-fold and 3.5-fold in therapeutic efficacy at 72 and 96 h, respectively, when p38α was deficient. Similar effects were obtained with dasatinib treatment (Figure 5) and another BCR-ABL-positive KBM5 (Figure 6 and Figure 7). These results suggest a potential novel strategy in providing a more effective and safer treatment for CML by combining TKIs, imatinib or dasatinib, and specific p38α inhibition.

The p38 MAPK family is a group of versatile serine/threonine kinases. The four members of p38 MAPKs (i.e., p38α, p38β, p38γ, and p38δ) exhibit high sequence homology and either overlap or have distinct physiological and pathological roles. Dysregulation of p38 activity is critically linked to the pathogenesis of hematopoietic disease, rheumatoid arthritis (RA), chronic obstructive pulmonary disease (COPD), and malignancy [17]. Since development of inhibitors with high specificity, particularly among the members of the sub-family, is very challenging, elucidation of the role of each isozyme is sometimes hampered. Due to the important role of p38α in pathogenesis, several highly specific p38α inhibitors have been discovered including TAK715. TAK715 displays high selectivity with an IC_50_ of 7.1 and 200 nM toward p38α and p38β, respectively [28]. In this study, we used either specific gene knockdown or a specific pharmacological inhibitor to provide unambiguous evidence discriminating the effect of the α and β isozymes on imatinib-mediated therapeutic efficacy (Figure 4 and Figure 8). This study unveiled the importance of isotype-specific intervention in enhancing therapeutic efficacy of imatinib.

Imatinib and dasatinib exert their therapeutic effects by targeting BCR-ABL, blocking downstream signaling and, thus, suppressing cell growth and inducing apoptosis [7,8]. These drugs may likely activate other pathways that play roles in promoting cell survival and cell proliferation due to the off-target effects. These unexpected effects may thus counteract the therapeutic efficacy of these drugs. We further searched the CLUE database, analyzed gene expression profiles with the TOUCHSTONE function, and unveiled that the gene signature of p38α overexpression exhibited a marked similarity with the gene signatures of imatinib and dasatinib [37] (Supplementary Materials Table S1), suggesting the possibility that imatinib and dasatinib may activate p38α signaling which has pro-survival and pro-proliferation effects. Together with the observation that imatinib and dasatinib activate the p38 MAPK pathway [20,21,35,36], a hypothesized model is shown in Supplementary Materials Figure S2. This provides an explanation for the significant enhancement of the therapeutic efficacy of imatinib and dasatinib upon knockdown or inhibition of p38α MAPK.

Taken together, our study using either specific gene knockdown or a specific pharmacological inhibitor provides strong evidence that p38α is a potential valuable target for combinational therapy to enhance the therapeutic efficacy of tyrosine kinase inhibitors, imatinib and dasatinib, toward CML patients. Imatinib is also used to treat BCR-ABL-positive ALL (acute lymphoblast leukemia) patients. Our findings are also potentially applicable for ALL patients in addition to CML patients.

## 4. Materials and Methods

### 4.1. Cell Culture

The human chronic myelogenous leukemia (CML) K562 cells were purchased from BCRC (Bioresource Collection and Research Center, Hsinchu, Taiwan) and cultured in RPMI1640 media containing 10% FBS as described [38]. KBM5 cells were obtained from Bing E. Carter (The University of Texas MD Anderson Cancer Center, Houston, TX, USA) and cultured in IMDM media containing 10% FBS. The pLKO.1 puro-based shRNAs specific to p38α and p38β were purchased from National RNAi Core Facility, Taiwan [22]. Transfection of K562 and KBM5 cells was performed using Lipofectamine^TM^ 2000 Reagent (Invitrogen, Carlsbad, CA, USA) and electroporation (Gene Pulser Xcell^TM^, Bio-Rad, Hercules, CA, USA), respectively. Stable clones of gene knockdown were selected in the presence of puromycin (0.5 μg/mL, Calbiochem, San Diego, CA, USA) for K562 cells or puromycin (0.15 µg/mL) for KMB5 cells.

### 4.2. Drug Treatment and Cytotoxicity Assay 

To examine the effects of therapeutic agents, K562 and KBM5 cells clones were seeded (8 × 10^4^ cells/35 mm dish) in regular media supplemented with 10% FBS for 24 h before the addition of drugs. Imatinib (SC-202180, Santa Cruz Biotechnology, Dallas, TX, USA) and dasatinib (SC-358114, Santa Cruz Biotechnology, Dallas, TX, USA) were dissolved in sterile distilled water, diluted to the desired concentrations, and added directly into media. The cells were then incubated at 37 °C in a CO_2_ incubator for periods as indicated. Cell numbers were counted using a hemocytometer under a light microscope. Dead cells were determined by trypan blue exclusion assay as described [24]. Briefly, an equal amount of cell suspension was mixed with trypan blue (0.4% in PBS), and cells were examined under a light microscope. Trypan blue can enter cells only when the cell membrane is disrupted. Therefore, the dead cells are stained blue and the viable cells remain clear under a light microscope. Total cell numbers represent the sum of dead cells and viable cells. The total viable cell numbers after drug treatment represent a combined outcome of cell death and growth suppression and were used as an overall measurement of therapeutic efficacy. The ratio of total viable cells between parental and knockdown cells is expressed as a fold increase.

To test the effect of specific p38α inhibitor, TAK715 (10 mM stock in DMSO) (HY-10456, MedChemExpress, Monmouth Junction, NJ, USA) was added into cells 1 h prior to the addition of imatinib or dasatinib. Cells were collected at different time points and examined.

### 4.3. Flow Cytometric Analysis

For cell cycle analysis, K562 parental and p38α KD1 cells (2 × 10^5^ cells/60 mm dish) were seeded in culture media for 24 h and then treated with various concentrations of imatinib for 48 or 72 h. Cells were collected, washed, and fixed in 70% ethanol at −20 °C overnight. Cells were gently washed, treated with RNase (0.5 mg/mL) at 37 °C for 15 min, and stained with propidium iodide (50 µg/mL) in the dark for 1 h at room temperature. Cells were filtered with cell strainer (35 µM, BD Falcon, Franklin Lakes, NJ, USA) and subjected to analysis by flow cytometry using the FACSCanto Flow Cytometer (BD Biosciences, Franklin Lakes, NJ, USA) equipped with FLOWJO software.

### 4.4. Western Blotting

Cells were seeded (4 × 10^5^) in a 100 mm dish. After drug treatment, cells were collected, washed, and lysed in RIPA buffer (i.e., 150 mM NaCl, 10 mM Tris, pH7.4, 1% Triton X-100, 0.1% SDS, 1% Na-deoxycholate, 5 mM EDTA, 1 mM PMSF, 10 μg/mL leupeptin, 10 μg/mL aprotinin, and 10 μg/mL pepstatin). Proteins were fractionated by SDS-PAGE followed by transfer to nitrocellulose membranes and subjected to detection using antibodies. The primary antibodies used were anti-caspase-3 (9662, 1:500, Cell Signaling, Danvers, MA, USA), anti-cleaved PARP (9541, 1:750, Cell Signaling, Danvers, MA, USA), anti-p38 (9212, 1:1000, Cell Signaling, Danvers, MA, USA), and anti-GAPDH (G9545, 1:10,000, Sigma–Aldrich, Burlington, MA, USA)). The secondary antibodies used were anti-mouse (Sigma–Aldrich, Burlington, MA, USA) or anti-rabbit (GeneTex, Irvine, CA, USA) IgG linked-horseradish peroxidase. The band intensity of cleaved PARP and caspase proteins in each lane was normalized to the band intensity of the corresponding GAPDH first, and the number of K562 at 0 h was 1.0.

### 4.5. Statistical Analysis

Data are presented as the means ± SEM. Statistical significance was determined using the Student’s *t*-test. A *p*-value < 0.05 was considered statistically significant. All experiments were performed at least three times independently.

## Supplementary Materials

The following are available online at https://www.mdpi.com/article/10.3390/ijms222212573/s1.

## References

[1] Ian C., Paul W. (2006). Design and development of signal transduction inhibitors for cancer treatment: Experience and challenges with kinase targets. Curr. Signal. Transduct. Ther..

[2] Davis M.I., Hunt J.P., Herrgard S., Ciceri P., Wodicka L.M., Pallares G., Hocker M., Treiber D.K., Zarrinkar P.P. (2011). Comprehensive analysis of kinase inhibitor selectivity. Nat. Biotechnol..

[3] Bayat Mokhtari R., Homayouni T.S., Baluch N., Morgatskaya E., Kumar S., Das B., Yeger H. (2017). Combination therapy in combating cancer. Oncotarget.

[4] Groffen J., Stephenson J.R., Heisterkamp N., de Klein A., Bartram C.R., Grosveld G. (1984). Philadelphia chromosomal breakpoints are clustered within a limited region, bcr, on chromosome 22. Cell.

[5] Sawyers C.L. (1999). Chronic myeloid leukemia. N. Engl. J. Med..

[6] Ferdinand R., Mitchell S.A., Batson S., Tumur I. (2012). Treatments for chronic myeloid leukemia: A qualitative systematic review. J. Blood Med..

[7] Quintas-Cardama A., Kantarjian H., Cortes J. (2007). Flying under the radar: The new wave of BCR-ABL inhibitors. Nat. Rev. Drug Discov..

[8] Cilloni D., Saglio G. (2012). Molecular pathways: BCR-ABL. Clin. Cancer Res..

[9] Kantarjian H.M., Giles F., Quintas-Cardama A., Cortes J. (2007). Important therapeutic targets in chronic myelogenous leukemia. Clin. Cancer Res..

[10] An X., Tiwari A.K., Sun Y., Ding P.R., Ashby C.R., Chen Z.S. (2010). BCR-ABL tyrosine kinase inhibitors in the treatment of Philadelphia chromosome positive chronic myeloid leukemia: A review. Leuk. Res..

[11] Freireich E.J., Wiernik P.H., Steensma D.P. (2014). The leukemias: A half-century of discovery. J. Clin. Oncol..

[12] Bhamidipati P.K., Kantarjian H., Cortes J., Cornelison A.M., Jabbour E. (2013). Management of imatinib-resistant patients with chronic myeloid leukemia. Ther. Adv. Hematol..

[13] Hochhaus A., Baccarani M., Silver R.T., Schiffer C., Apperley J.F., Cervantes F., Clark R.E., Cortes J.E., Deininger M.W., Guilhot F. (2020). European LeukemiaNet 2020 recommendations for treating chronic myeloid leukemia. Leukemia.

[14] Vener C., Banzi R., Ambrogi F., Ferrero A., Saglio G., Pravettoni G., Sant M. (2020). First-line imatinib vs. second- and third-generation TKIs for chronic-phase CML: A systematic review and meta-analysis. Blood Adv..

[15] Weisberg E., Manley P.W., Cowan-Jacob S.W., Hochhaus A., Griffin J.D. (2007). Second generation inhibitors of BCR-ABL for the treatment of imatinib-resistant chronic myeloid leukaemia. Nat. Rev. Cancer.

[16] Kim E.K., Choi E.J. (2010). Pathological roles of MAPK signaling pathways in human diseases. Biochim. Biophys. Acta.

[17] Cuenda A., Rousseau S. (2007). p38 MAP-kinases pathway regulation, function and role in human diseases. Biochim. Biophys. Acta.

[18] Canovas B., Nebreda A.R. (2021). Diversity and versatility of p38 kinase signalling in health and disease. Nat. Rev. Mol. Cell Biol..

[19] Cuadrado A., Nebreda A.R. (2010). Mechanisms and functions of p38 MAPK signalling. Biochem. J..

[20] Dong Y., Xiong M., Duan L., Liu Z., Niu T., Luo Y., Wu X., Xu C., Lu C. (2014). H2AX phosphorylation regulated by p38 is involved in Bim expression and apoptosis in chronic myelogenous leukemia cells induced by imatinib. Apoptosis.

[21] Parmar S., Katsoulidis E., Verma A., Li Y., Sassano A., Lal L., Majchrzak B., Ravandi F., Tallman M.S., Fish E.N. (2004). Role of the p38 mitogen-activated protein kinase pathway in the generation of the effects of imatinib mesylate (STI571) in BCR-ABL-expressing cells. J. Biol. Chem..

[22] Jacquel A., Colosetti P., Grosso S., Belhacene N., Puissant A., Marchetti S., Breittmayer J.P., Auberger P. (2007). Apoptosis and erythroid differentiation triggered by Bcr-Abl inhibitors in CML cell lines are fully distinguishable processes that exhibit different sensitivity to caspase inhibition. Oncogene.

[23] Chang Y.I., Hua W.K., Yao C.L., Hwang S.M., Hung Y.C., Kuan C.J., Leou J.S., Lin W.J. (2010). Protein-arginine methyltransferase 1 suppresses megakaryocytic differentiation via modulation of the p38 MAPK pathway in K562 cells. J. Biol. Chem..

[24] Liu M.Y., Hua W.K., Chen C.J., Lin W.J. (2020). The MKK-Dependent Phosphorylation of p38alpha Is Augmented by Arginine Methylation on Arg49/Arg149 during Erythroid Differentiation. Int. J. Mol. Sci..

[25] Hua W.K., Chang Y.I., Yao C.L., Hwang S.M., Chang C.Y., Lin W.J. (2013). Protein arginine methyltransferase 1 interacts with and activates p38alpha to facilitate erythroid differentiation. PLoS ONE.

[26] Shah N.P., Kasap C., Weier C., Balbas M., Nicoll J.M., Bleickardt E., Nicaise C., Sawyers C.L. (2008). Transient potent BCR-ABL inhibition is sufficient to commit chronic myeloid leukemia cells irreversibly to apoptosis. Cancer Cell.

[27] Beran M., Pisa P., O’Brien S., Kurzrock R., Siciliano M., Cork A., Andersson B.S., Kohli V., Kantarjian H. (1993). Biological properties and growth in SCID mice of a new myelogenous leukemia cell line (KBM-5) derived from chronic myelogenous leukemia cells in the blastic phase. Cancer Res..

[28] Miwatashi S., Arikawa Y., Kotani E., Miyamoto M., Naruo K., Kimura H., Tanaka T., Asahi S., Ohkawa S. (2005). Novel inhibitor of p38 MAP kinase as an anti-TNF-alpha drug: Discovery of N-[4-[2-ethyl-4-(3-methylphenyl)-1,3-thiazol-5-yl]-2- pyridyl]benzamide (TAK-715) as a potent and orally active anti-rheumatoid arthritis agent. J. Med. Chem..

[29] Menzies A.M., Long G.V. (2014). Dabrafenib and trametinib, alone and in combination for BRAF-mutant metastatic melanoma. Clin. Cancer Res..

[30] Larkin J., Ascierto P.A., Dreno B., Atkinson V., Liszkay G., Maio M., Mandala M., Demidov L., Stroyakovskiy D., Thomas L. (2014). Combined vemurafenib and cobimetinib in BRAF-mutated melanoma. N. Engl. J. Med..

[31] Robinson K.W., Sandler A.B. (2013). The role of MET receptor tyrosine kinase in non-small cell lung cancer and clinical development of targeted anti-MET agents. Oncologist.

[32] Pao W., Chmielecki J. (2010). Rational, biologically based treatment of EGFR-mutant non-small-cell lung cancer. Nat. Rev. Cancer.

[33] Oaxaca D.M., Yang-Reid S.A., Ross J.A., Rodriguez G., Staniswalis J.G., Kirken R.A. (2016). Sensitivity of imatinib-resistant T315I BCR-ABL CML to a synergistic combination of ponatinib and forskolin treatment. Tumor Biol..

[34] Carter B.Z., Mak P.Y., Mu H., Zhou H., Mak D.H., Schober W., Leverson J.D., Zhang B., Bhatia R., Huang X. (2016). Combined targeting of BCL-2 and BCR-ABL tyrosine kinase eradicates chronic myeloid leukemia stem cells. Sci. Transl. Med..

[35] Kohmura K., Miyakawa Y., Kawai Y., Ikeda Y., Kizaki M. (2004). Different roles of p38 MAPK and ERK in STI571-induced multi-lineage differentiation of K562 cells. J. Cell. Physiol..

[36] Dumka D., Puri P., Carayol N., Lumby C., Balachandran H., Schuster K., Verma A.K., Terada L.S., Platanias L.C., Parmar S. (2009). Activation of the p38 Map kinase pathway is essential for the antileukemic effects of dasatinib. Leuk. Lymphoma.

[37] Subramanian A., Narayan R., Corsello S.M., Peck D.D., Natoli T.E., Lu X., Gould J., Davis J.F., Tubelli A.A., Asiedu J.K. (2017). A Next Generation Connectivity Map: L1000 Platform and the First 1,000,000 Profiles. Cell.

[38] Liu M.Y., Hua W.K., Chiou Y.Y., Chen C.J., Yao C.L., Lai Y.T., Lin C.H., Lin W.J. (2020). Calcium-dependent methylation by PRMT1 promotes erythroid differentiation through the p38alpha MAPK pathway. FEBS Lett..

