# Supplementary material for: Perturbation of p38α MAPK as a Novel Strategy to Effectively Sensitize Chronic Myeloid Leukemia Cells to Therapeutic BCR-ABL Inhibitors
Source: Int J Mol Sci. 2021 Nov 22;22(22):12573. doi: 10.3390/ijms222212573 (PMC8623086; doi:10.3390/ijms222212573)
Supplement: Supplementary file 1 [file ijms-22-12573-s001.zip › Supplementary figure legends Revision.pdf]

Supplementary figure legends :

Table S1. Similarity of gene signature between p38α overexpression and TKI treatment.

Gene expression profiles in CLUE database were analyzed. Similarity was presented as summary score.

Figure S1. The p38α-knockdown K562 cell clone KD11 exhibits a higher sensitivity to imatinib-induced cell cytotoxicity.

The transcripts of p38α in K562 cells were knocked down using specific shRNAs. The protein levels p38α in KD11 stable clone were examined by Western blotting (A, upper). The cell growth of parental K562 and knockdown KD11 cell clones were examined by using hemocytometer counting under a microscope (A, bottom). Dead cells were identified by trypan blue exclusion. The cells were treated with imatinib (0.3 μM) and cell viability was examined (B). The sensitivity to the cytotoxic effect of imatinib was significantly increased in the knockdown KD11 cells. All results shown are representatives of three independent experiments. Cell number and viability are presented as mean ± S.E. of three repeats. *, $p < 0.05$, **, $p< 0.01$ and ***, $p < 0.005$.

Figure S2. Illustration of the hypothetic model for the combined effects of p38α and BCR-ABL inhibition.

The BCR-ABL oncogene inhibits apoptosis and growth suppression and results in uncontrolled growth, poor differentiation and malignancy (left panel). TKIs (imatinib and dasatinib) inhibit the kinase activity of BCR-ABL and reverse its suppression on growth and apoptosis. Therefore, cells regain the ability of growth control, apoptosis and differentiation. Meanwhile, imatinib (TKIs) may activate p38α which suppresses apoptosis and growth inhibition (middle panel). The outcome of these effects together impedes the overall therapeutic efficacy of TKIs. Upon p38α suppression by either gene knockdown or pharmacological inhibition, the TKIs exert a much better efficacy at a lower concentration (right panel).
