# Supplementary material for: Perturbation of p38α MAPK as a Novel Strategy to Effectively Sensitize Chronic Myeloid Leukemia Cells to Therapeutic BCR-ABL Inhibitors
Source: Int J Mol Sci. 2021 Nov 22;22(22):12573. doi: 10.3390/ijms222212573 (PMC8623086; doi:10.3390/ijms222212573)
Supplement: Supplementary file 1 [file ijms-22-12573-s001.zip › Supplementary Materials Rivision.pdf]

Similarity in gene signature between p38α overexpression and TKI treatment		
Compound	Description	Summary score
Imatinib	BCR-ABL kinase inhibitor	84.98
Dasatinib	BCR-ABL kinase inhibitor	84.23

Supplementary Table S1

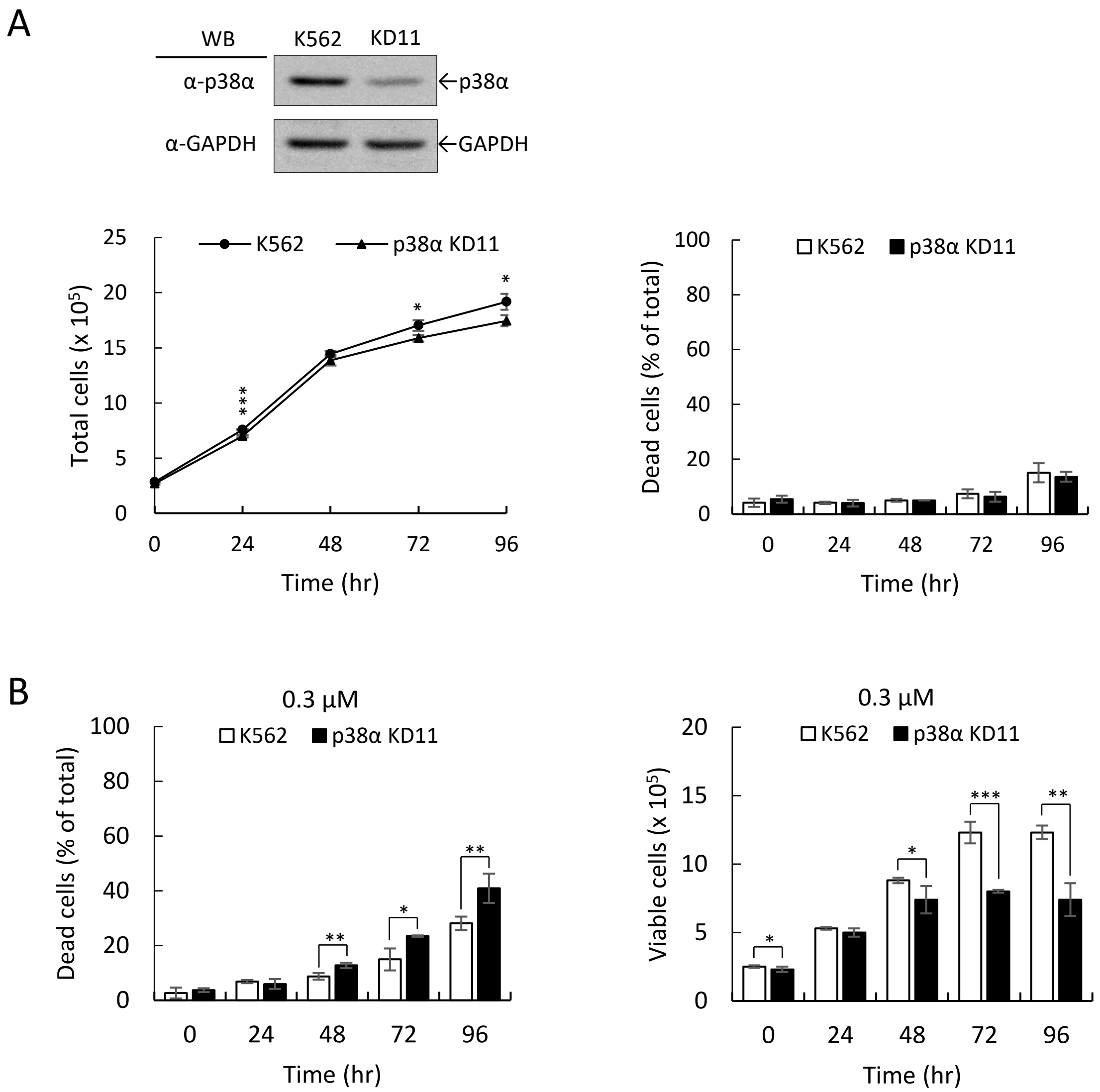

Supplementary Figure S1

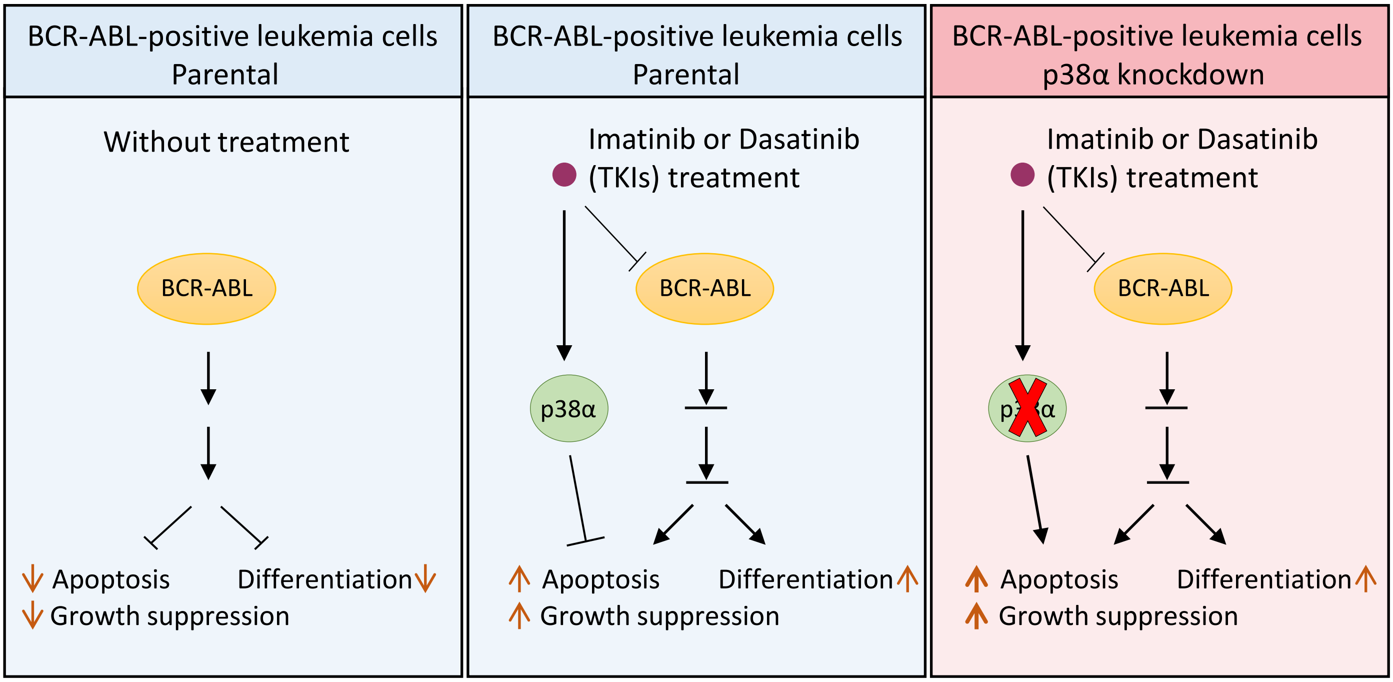

Supplementary Figure S2
